# CHOP upregulation and dysregulation of the mature form of the SNAT2 amino acid transporter in the placentas from small for gestational age newborns

**DOI:** 10.1186/s12964-023-01352-5

**Published:** 2023-11-13

**Authors:** Emma Barroso, Marta Díaz, Ana Cristina Reguera, Mona Peyman, Jesús Balsinde, Javier Jurado-Aguilar, Meijian Zhang, Adel Rostami, Xavier Palomer, Lourdes Ibáñez, Manuel Vázquez-Carrera

**Affiliations:** 1 Unitat de Farmacologia, Facultat de Farmàcia I Ciències de L’Alimentació, Institute of Biomedicine of the University of Barcelona (IBUB), University of Barcelona, Av. Joan XXIII 27-31, 08028 Barcelona, Spain; 2 Spanish Biomedical Research Center in Diabetes and Associated Metabolic Diseases (CIBERDEM)-Instituto de Salud Carlos III, Madrid, Spain; 3 Pediatric Research Institute-Hospital Sant Joan de Déu, Esplugues de Llobregat, Spain; 4 Endocrinology, Pediatric Research Institute, Sant Joan de Déu Children’s Hospital, Barcelona, Esplugues Spain; 5 Instituto de Biología y Genética Molecular, Consejo Superior de Investigaciones Científicas, Valladolid, Spain

**Keywords:** Placenta, CHOP, SNAT2, mTORC1, ER stress, GADD34

## Abstract

**Background:**

The placentas from newborns that are small for gestational age (SGA; birth weight < -2 SD for gestational age) may display multiple pathological characteristics. A key determinant of fetal growth and, therefore, birth weight is placental amino acid transport, which is under the control of the serine/threonine kinase mechanistic target of rapamycin (mTOR). The effects of endoplasmic reticulum (ER) stress on the mTOR pathway and the levels of amino acid transporters are not well established.

**Methods:**

Placentas from SGA and appropriate for gestational age (AGA) newborns and the human placental BeWo cell line exposed to the ER stressor tunicamycin were used.

**Results:**

We detected a significant increase in the levels of C/EBP homologous protein (CHOP) in the placentas from SGA newborns compared with those from AGA newborns, while the levels of other ER stress markers were barely affected. In addition, placental mTOR Complex 1 (mTORC1) activity and the levels of the mature form of the amino acid transporter sodium-coupled neutral amino acid transporter 2 (SNAT2) were also reduced in the SGA group. Interestingly, CHOP has been reported to upregulate growth arrest and DNA damage-inducible protein 34 (GADD34), which in turn suppresses mTORC1 activity. The GADD34 inhibitor guanabenz attenuated the increase in CHOP protein levels and the reduction in mTORC1 activity caused by the ER stressor tunicamycin in the human placental cell line BeWo, but it did not recover mature SNAT2 protein levels, which might be reduced as a result of defective glycosylation.

**Conclusions:**

Collectively, these data reveal that GADD34A activity and glycosylation are key factors controlling mTORC1 signaling and mature SNAT2 levels in trophoblasts, respectively, and might contribute to the SGA condition.

Video Abstract

**Supplementary Information:**

The online version contains supplementary material available at 10.1186/s12964-023-01352-5.

## Background

Newborns with a birth weight for gestational age less than -2 SD are considered to be small for gestational age (SGA) [1]. SGA infants, particularly those who experience a rapid and significant catch-up in weight, have an increased risk of developing metabolic disorders later in life, including visceral obesity, hypertension, insulin resistance, type 2 diabetes, and cardiovascular disease [2]. The SGA condition has been associated with multiple placental pathological characteristics [3–5]. A feature of placental pathophysiology is endoplasmic reticulum (ER) stress, which is considered a target for pregnancy complications [6, 7]. The ER has key functions within cells, including the synthesis, folding, and transport of proteins. The accumulation of misfolded and unfolded proteins in the ER lumen disrupts the homeostasis of this organelle and leads to ER stress, which activates the unfolded protein response (UPR) [8]. This is an adaptive response that involves the activation of a signaling pathway in order to restore the folding capacity. The UPR involves the activation of three transmembrane proteins: inositol-requiring enzyme 1 (IRE-1), activating transcription factor 6 (ATF6), and protein kinase RNA (PKR)-like ER kinase (PERK). PERK phosphorylates eukaryotic initiation factor 2α (eIF2α) and attenuates protein translation, thereby reducing the amounts of new proteins entering the ER lumen. However, if the UPR cannot restore ER homeostasis, apoptosis is induced by the PERK-eIF2α pathway and the subsequent increase in ATF4 activity, which upregulates the expression of C/EBP homologous protein (CHOP) and growth arrest and DNA damage-inducible protein 34 (GADD34). Remarkably, CHOP upregulation has been associated with compromised placental development and function in vivo and in vitro [9–11].

A key determinant of fetal growth and, therefore, birth weight is placental nutrient transfer. This is highly dependent on the levels of nutrient transporters in the epithelium of the placenta, the syncytiotrophoblast [12]. A reduction in the placental activity of system A, a sodium-dependent transporter mediating the uptake of non-essential amino acids, has been associated with decreased birth weight in humans [13, 14] and in animal models [15, 16]. System A includes several subtypes of sodium-coupled neutral amino acid transporters (SNATs) with similar substrate specificities: SNAT1, SNAT2, and SNAT4 [17]. Similar reductions have been reported in the levels of transporters of essential amino acids, such as system L, which includes L-type amino acid transporter 1 (LAT1) and LAT2 [18, 19]. Trophoblast system A and system L amino acid transporter activities are under the control of the serine/threonine kinase mechanistic target of rapamycin (mTOR) [20]. mTOR forms two distinct complexes, mTORC1 and mTORC2. The function of mTORC1 is mediated by the phosphorylation of downstream targets, mainly eukaryotic initiation factor 4E-binding protein 1 (4E-BP1) and p70 S6 kinase (S6K), the latter catalyzing the phosphorylation of ribosomal protein (RP)S6. Activation of mTORC2 promotes the phosphorylation of protein kinase B (Akt), protein kinase Cα (PKCα), and serum and glucocorticoid-regulated kinase 1 (SGK1). Notably, the silencing of mTORC1 and/or mTORC2 results in a marked inhibition of trophoblast system A and system L amino acid transporter activities [20], thereby indicating that the inhibition of placental mTOR is involved in decreased placental amino acid uptake and fetal growth. However, little is known about how the activation of the ER stress/UPR process results in a reduction in mTOR signaling and in the levels of amino acid transporters in the placenta and trophoblasts.

Here, we show that the ER stress marker CHOP is upregulated in the placentas from SGA newborns, which is accompanied by reduced mTORC1 activity and a significant decrease in the mature form of the amino acid transporter SNAT2. Interestingly, inhibition of GADD34A in a human placental cell line by the ER stressor tunicamycin attenuates the reduction in mTORC1 activity, but does not recover mature SNAT2 protein levels, which might be reduced as a result of defective glycosylation. These findings suggest that the dysregulation of CHOP and of the processing/maturation of SNAT2 may contribute to the SGA condition.

## Methods

### Reagents

Guanabenz and tunicamycin were purchased from Sigma-Aldrich (Madrid, Spain).

### Study population

The study cohort consisted of 40 mother-newborn pairs recruited at the Hospital Sant Joan de Déu of Barcelona (Spain) from a prenatal cohort study of mothers and infants. Twenty infants were born AGA (10 girls, 10 boys) and 20 SGA (10 girls, 10 boys). The inclusion criteria were: (i) infants born at term (37–42 weeks) from singleton pregnancies and with a birth weight between -1.0 and + 1.0 SD (range 2.9–3.8 kg) for AGA and below -2 SD (range 1.9–2.6 kg) for SGA; (ii) placenta collected at delivery; and (iii) written informed consent obtained in the third trimester of pregnancy. The exclusion criteria were: (i) maternal disease (hypertension, preeclampsia, gestational diabetes, or preexisting type 1 and type 2 diabetes mellitus), alcohol abuse or drug addiction; and (ii) fetal malformations or complications at birth. The SGA babies included in the present study had normal umbilical flow indices, and none of them had oligohydramnios or neonatal complications.

The study was approved by the Institutional Review Board of the Hospital Sant Joan de Déu at the University of Barcelona.

### Clinical, endocrine-metabolic and body composition assessments

Information on maternal age at conception, height, pregestational weight, and gestational weight gain were retrieved from the mother’s clinical records. Gestational age was calculated from the last menses and was confirmed by a first-trimester ultrasound.

The weight and length of the newborns were measured in the delivery room and transformed into Z-scores according to country and sex-specific growth charts [21]. Blood samples were obtained at birth from the umbilical cord, before placental separation.

Serum glucose levels were measured with the glucose oxidase method. Insulin and insulin-like growth factor-1 (IGF-1) were assessed by immunechemiluminescence (DPC, IMMULITE 2500, Siemens, Erlangen, Germany), with the intra- and inter-assay coefficient of variation (CVs) being < 10%.

Homeostatic model assessment for insulin resistance (HOMA-IR) was calculated as fasting insulin (mU/L) x fasting glucose (mmol/L)/22.5. Circulating high molecular weight (HMW)-adiponectin was measured with a specific enzyme-linked immunosorbent assay (R&D Systems, Minneapolis, USA), with the intra- and inter-assay CVs being < 9%. Glucagon-like peptide-1 (GLP-1) was assessed by an enzyme-linked immunosorbent assay (Millipore, Billerica, MA, USA). The antibody pair in the assay binds to GLP-1 (7–36) and (9–36) and has no cross-reactivity with GLP-2, GIP, glucagon or oxyntomodulin. The intra-assay and inter-assay CVs were < 2% and < 10%, respectively, and the detection limit was 1.5 pM.

Body composition was assessed at the age of 15 days by dual X-ray absorciometry with a Lunar Prodigy system coupled to Lunar software (Lunar Corp, Madison, WI, USA) adapted for infants. CVs were < 3% for fat and lean mass [22].

### Placenta collection

Placentas were collected after childbirth in the delivery room and weighed immediately. Placental tissue encompassing the decidua and the upper side of the chorionic villous proximal to the decidua was dissected to obtain placental maternal biopsies. Three pieces of 1-cm^3^ cuboidal sections were collected from the maternal side after removal of the amniotic and chorionic layers. Placental samples were washed three times with physiological saline to remove all maternal blood and immediately frozen in liquid nitrogen and stored at − 80 °C until analysis. The personnel always wore face masks and sterile gloves and used a sterile scalpel and instruments.

For the studies of mRNA and protein expression in the placentas from AGA and SGA infants, only girls were selected (as specified in the Results section).

### Cell culture

BeWo cells (kindly donated by Dr. Vicente Andreu Fernández from the Universidad Internacional de Valencia, Valencia) were cultured in Ham’s F12 medium (Gibco-Invitrogen) supplemented with 10% fetal bovine serum (FBS) (Sigma-Aldrich) and 1% penicillin–streptomycin (Gibco-Invitrogen). Cells were seeded and, 72 h later, differentiated into syncytiotrophoblasts by incubation with 40 μM forskolin (Santa Cruz Biotechnology) for 48 h. Once differentiated, cells were treated with 0.1 μg/ml of tunicamycin (TM) (Sigma-Aldrich) and 5 μM guanabenz (GB) (Sigma-Aldrich), which was added 1 h before tunicamycin, for 24 h.

### Reverse transcription-polymerase chain reaction and quantitative polymerase chain reaction

Isolated RNA was reverse transcribed to obtain 1 μg of complementary DNA (cDNA) using Random Hexamers (Thermo Scientific), 10 mM deoxynucleotide (dNTP) mix, and the reverse transcriptase enzyme derived from the Moloney murine leukemia virus (MMLV, Thermo Fisher). The experiment was run in a thermocycler (BioRad) and consisted of a program with different steps and temperatures: 65 °C for 5 min, 4 °C for 5 min, 37 °C for 2 min, 25 °C for 10 min, 37 °C for 50 min, and 70 °C for 15 min. The relative levels of specific mRNAs were assessed by real-time RT-PCR in a mini 48-well T100™ thermal cycler (Bio-Rad), using the SYBR Green Master Mix (Applied Biosystems), as previously described [23]. Briefly, samples had a final volume of 20 μl, containing 20 ng of total cDNA, 0.9 μM of the primer mix, and 10 μl of 2 × SYBR Green Master Mix. The thermal cycler protocol for real-time PCR included a first step of denaturation at 95 °C for 10 min followed by 40 repeated cycles of 95 °C for 15 s, 60 °C for 30 s, and 72 °C for 30 s for denaturation, primer annealing, and amplification, respectively. Primer sequences were designed using the Primer-BLAST tool (NCBI), based on the full mRNA sequences to find the optimal primers for amplification, and evaluated with the Oligo-Analyzer Tool (Integrated DNA Technologies) to ensure an optimal melting temperature (Tm) and avoid the formation of homo/heterodimers or non-specific structures that can interfere with the interpretation of the results. The primer sequences were designed specifically to span the junction between the exons. The primer sequences used were: *CHOP*, 5’-GGAAATGAAGAGGAAGAATCAAAAAT-3’ and 5’-GTTCTGGCTCCTCCTCAGTCA-3’; *GRP78/BiP*, 5’- ACTATTGCTGGCCTAAATGTTATGAG-3’ and 5’-TTATCCAGGCCATAAGCAATAGC-3’; *SLC7A5*/*LAT1*, 5’-CAGTACATCGTGGCCCTGGT-3’ and 5’-TGAGCAGCAGCACGCAGAG-3’; *SLC38A1*/*SNAT1*, 5’-GTGTATGCTTTACCCACCATTGC-3’ and 5’- GCACGTTGTCATAGAATGTCAAGT-3’; *SLC38A2*/*SNAT2*, 5’- ACGAAACAATAAACACCACCTTAA-3’ and 5’-AGATCAGAATTGGCACAGCATA-3’; and *TBP*, 5’-CCACTCACAGACTCTCACAAC-3’ and 5’-CTGCGGTACAATCCCAGAACT-3’. Values were normalized to the expression levels of TATA-box-binding protein (TBP), and measurements were performed in triplicate. All changes in expression were normalized to the untreated control.

### Immunoblotting

The isolation of total protein extracts was performed as described elsewhere [24]. For the isolation of total cell membranes, cell suspensions or placenta tissues were resuspended in 3 ml of ice-cold buffer I (250 mM sucrose, 20 mM HEPES, 5 mM NaN_3_, 2 mM EGTA, 100 μM phenylmethylsulfonyl fluoride, 10 μM L-trans-epoxysuccinyl-leucylamido(4-guanidino)butane, 1 μM pepstatin A, and 1 μM leupeptin; pH 7.4) and homogenized. The resulting homogenate was centrifuged at 177,000 g for 1 h at 4 °C. The pellet, containing the total membranes, was resuspended in 50 μl of buffer I supplemented with a protease inhibitor and stored at -20 °C.

Immunoblotting was performed with antibodies against β-actin (Sigma, A5441), 4EBP1 (Cell Signalling, 9452), AKT (Cell Signalling, 9272), AMPKα (Cell Signalling, 2532), ATF4 (Santa Cruz Biotechnology, sc-200), ATF6 (Santa Cruz Biotechnology, sc-22799), BiP/GRP78 (Cell Signalling, 3183), CHOP (Cell Signalling, 2895), eIF2α (Cell Signalling, 9722), ERK1/2 (44/42 MAPK) (Cell Signalling, 9102), GADD34 (Cell Signalling, sc-46661), GAPDH (Millipore, MAB374), GRASP55 (Proteintech, 66,627–1-Ig), LAT1 (Cell Signalling, 5347), mTOR (Cell Signalling, 2972), Na–K-ATPase (Santa Cruz Biotechnology, sc-514614), NEDD4-L (Cell Signalling, sc-514954), phospho-4EBP1 Thr^37^/^46^ (Cell Signalling, 2855), phospho-AKT Ser^473^ (Cell Signalling, 9271), phospho-AMPKα Thr^172^ (Cell Signalling, 2531), phospho-ERK1/2 (44/42 MAPK) Thr^202^/Tyr^204^ (Cell Signalling, 9101), phospho-IRE Ser^724^ (Novus Biologicals, NB100-2323), phospho-mTOR Ser^2448^ (Santa Cruz Biotechnology, sc-101738), phospho-ribosomal protein S6 (Cell Signalling, 2211), ribosomal protein S6 (Cell Signalling, 2317), SNAT1 (Novus Biologicals, NBP-2–59311), SNAT2 (MBL, BMP081), and vinculin (Santa Cruz Biotechnology, sc-73614). Signal acquisition was conducted using the Bio-Rad ChemiDoc apparatus and the quantification of the immunoblot signal was performed with the Bio-Rad Image Lab software. The results for protein quantification were normalized to the levels of a control protein (GAPDH, β-actin, vinculin, or Na–K-ATPase) to avoid unwanted sources of variation.

### Statistical analysis

Results are expressed as the mean ± SEM. Significant differences were assessed by either Student’s t-test or one-way ANOVA, according to the number of groups compared, using the GraphPad Prism program (version 9.0.2) (GraphPad Software Inc., San Diego, CA, USA). When significant variations were found by ANOVA, Tukey’s post-hoc test for multiple comparisons was performed only if F achieved a *p* value < 0.05. Differences were considered significant at *p* < 0.05.

## Results

### Maternal and newborn characteristics

**Fig. 1:**
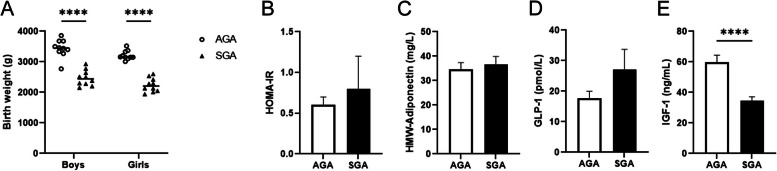
Selected anthropometric and endocrine-metabolic parameters in infants born appropriate (AGA) or small for gestational age (SGA). **A** Birth weight of newborns split by sex (*N* = 10). Serum HOMA-IR (**B**), serum high molecular weight (HMW)-adiponectin (**C**), GLP-1 (**D**) and circulating insulin-like growth factor 1 (IGF-1) (**E**) (*N* = 20). Data are presented as the mean ± SEM. **** p < 0.0001 *versus* the AGA group. *P*-values determined by two-tailed unpaired Student’s t-test

Table 1 shows the anthropometric parameters of the women and their newborns according to the birth weight groups. No significant differences were observed between the appropriate-for-gestational-age (AGA) and SGA groups regarding maternal age at conception, maternal height, and the pregestational weight gain, while the maternal weight, ponderal index and BMI were reduced in the SGA group (Table 1). As expected, caesarean sections were more frequent among SGA babies and SGA infants at birth showed reductions in the length of gestation, placental weight, birth weight, birth length and ponderal index (Table 1). No differences were found in birth weight when the data were separated by sex (Fig. 1A). Fat mass and lean mass were reduced in the SGA group compared with the AGA group, whereas no differences were observed for abdominal fat (Table 1). Homeostatic model assessment for insulin resistance (HOMA-IR), high molecular weight (HMW)-adiponectin, and glucagon-like peptide 1 (GLP-1) did not show differences between the AGA and SGA groups (Fig. 1B, C and D). However, insulin-like growth factor 1 (IGF-1) levels were significantly lower in the SGA group (Fig. 1E).

**Table 1:** Characteristics of the studied population

	AGA (*n* = 20)	SGA (*n* = 20)	*P* value
**Mothers**			
Age	33.8 ± 1.2	31.9 ± 1.2	0.266
Height (cm)	163.7 ± 1.1	160.2 ± 1.3	0.051
Weight (Kg)^a^	63.1 ± 2.6	54.1 ± 1.7	**0.005**
BMI (Kg/m^2^)	23.5 ± 0.8	21.0 ± 0.5	**0.016**
Ponderal index (Kg/m^3^)	14.3 ± 0.5	13.1 ± 0.3	**0.045**
Gestational weight gain (Kg)	15.0 ± 1.3	12.3 ± 0.8	0.084
**Newborns**			
Sex (% females)	50	50	
Gestational age (weeks)	39.8 ± 0.2	38.8 ± 0.4	**0.029**
Caesarean section (%)	5	35	**0.018**
Placental weight (g)	554 ± 13	474 ± 10	** < 0.0001**
Birth weight (Kg)	3.3 ± 0.1	2.3 ± 0.1	** < 0.0001**
Birth length (cm)	50.0 ± 0.4	46.1 ± 0.4	** < 0.0001**
Ponderal index (Kg/m^3^)	26.7 ± 0.5	24.0 ± 0.3	** < 0.0001**
**Endocrine-metabolic variables**			
HOMA-IR	0.6 ± 0.1	0.8 ± 0.4	0.515
IGF-1 (ng/mL)	59.6 ± 4.8	34.6 ± 2.5	** < 0.0001**
HMW-adiponectin (mg/L)	34.7 ± 2.7	36.7 ± 3.2	0.645
GLP-1 (pmol/L)	17.7 ± 2.3	27.0 ± 6.7	0.150
**Body composition (DXA)**^b^			
Fat mass (g)	617 ± 38	482 ± 37	**0.015**
Abdominal fat (g)	24.8 ± 2.7	18.1 ± 2.7	0.091
Lean mass (Kg)	3.1 ± 0.1	2.3 ± 0.1	** < 0.0001**

Results are mean ± sem

^a^Pre-gestational weight

^b^Assessed at 15 days of life

### SGA is associated with increased placental levels of CHOP and inhibition of the mTORC1 pathway

**Fig. 2:**
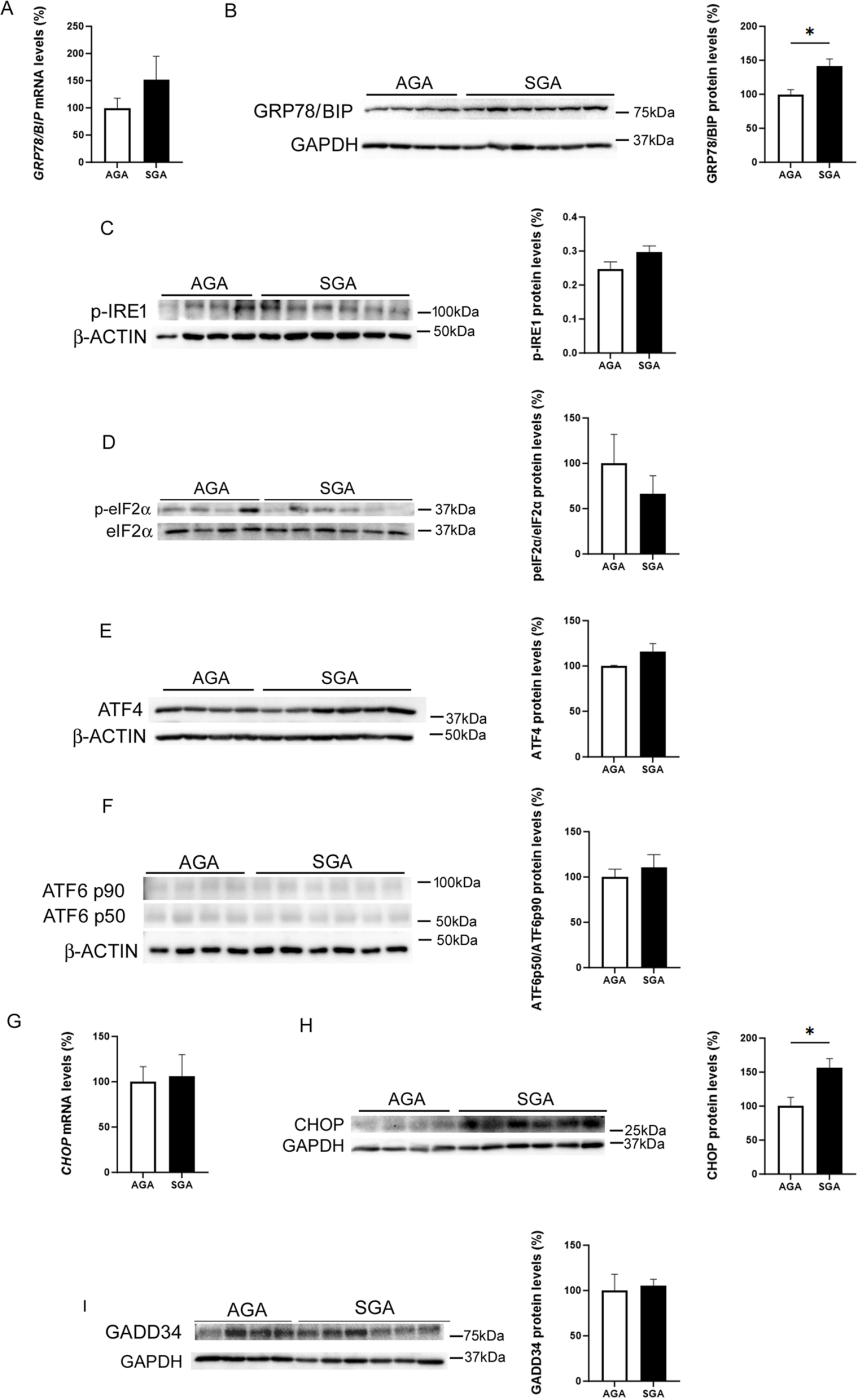
Placental CHOP protein levels are increased in SGA newborns. Placental GRP78/BiP mRNA (**A**) and protein (**B**) levels in the AGA and SGA groups. Placental cell lysate extracts were assayed via western blot analysis with antibodies against phosphorylated IRE (**C**), phosphorylated eIF2α (**D**), ATF4 (**E**) and ATF6 (**F**). Placental CHOP mRNA (**G**) and protein (**H**) levels in the AGA and SGA groups. **I** GADD34 protein levels. Data (*N* = 10) are presented as the mean ± SEM. * p < 0.05 *versus* the AGA group. *P*-values determined by two-tailed unpaired Student’s t-test

Since placental development differs between female and male newborns [25], we selected placental samples from newborn girls to examine the levels of proteins involved in ER stress. First, we assessed the protein levels of 78-kDa glucose-regulated protein (GRP78), which is also referred to as binding immunoglobulin protein (BiP). This protein is induced by ER stress and is a master regulator for this process through its role as a major ER chaperone with antiapoptotic properties, as well as its ability to control the activation of UPR signaling [8]. Placental GRP78 mRNA (Fig. 2A) and protein levels (Fig. 2B) were higher in the SGA than in the AGA group. Upon ER stress, GRP78 is released from the ER transmembrane, and IRE1, PERK, and ATF6 are activated. After the disassociation from GRP78, PERK dimerizes and undergoes autophosphorylation and activation. Activated PERK then phosphorylates eIF2α, which in turn increases the activity of ATF4, a transcription factor that upregulates the expression of CHOP. ATF6 is a large 90 kDa protein anchored at the ER membrane, which translocates to the Golgi apparatus in response to ER stress. Once in the Golgi, it is cleaved to release a smaller 50 kDa active protein, which enters the nucleus and acts as a transcription factor. No significant changes were observed in the levels of phospho-IRE1α and phospho-eIF2α (Fig. 2C, D). Likewise, ATF4 (Fig. 2E) or ATF6 (Fig. 2F) protein levels were not significantly different between the SGA and AGA groups. The expression of CHOP was not affected (Fig. 2G), while its protein levels were increased in the SGA group (Fig. 2H). Although *GADD34* transcription has been reported to be activated by ATF4 and CHOP [24], no significant changes were observed in its protein levels, which is likely to be the result of the reported transient increase in this protein under ER stress conditions [26] (Fig. 2I). Although ER stress has been associated with a reduction in the activity of AMP-activated protein kinase (AMPK) and the subsequent increase in the activity of extracellular signal-regulated kinase (ERK)1/2 [27], no differences were observed in the phosphorylated levels of AMPK and ERK1/2 between the AGA and SGA groups (Supplementary Fig. 1A, B), making it unlikely that these kinases were involved in the development of placental ER stress in the SGA group.

**Fig. 3:**
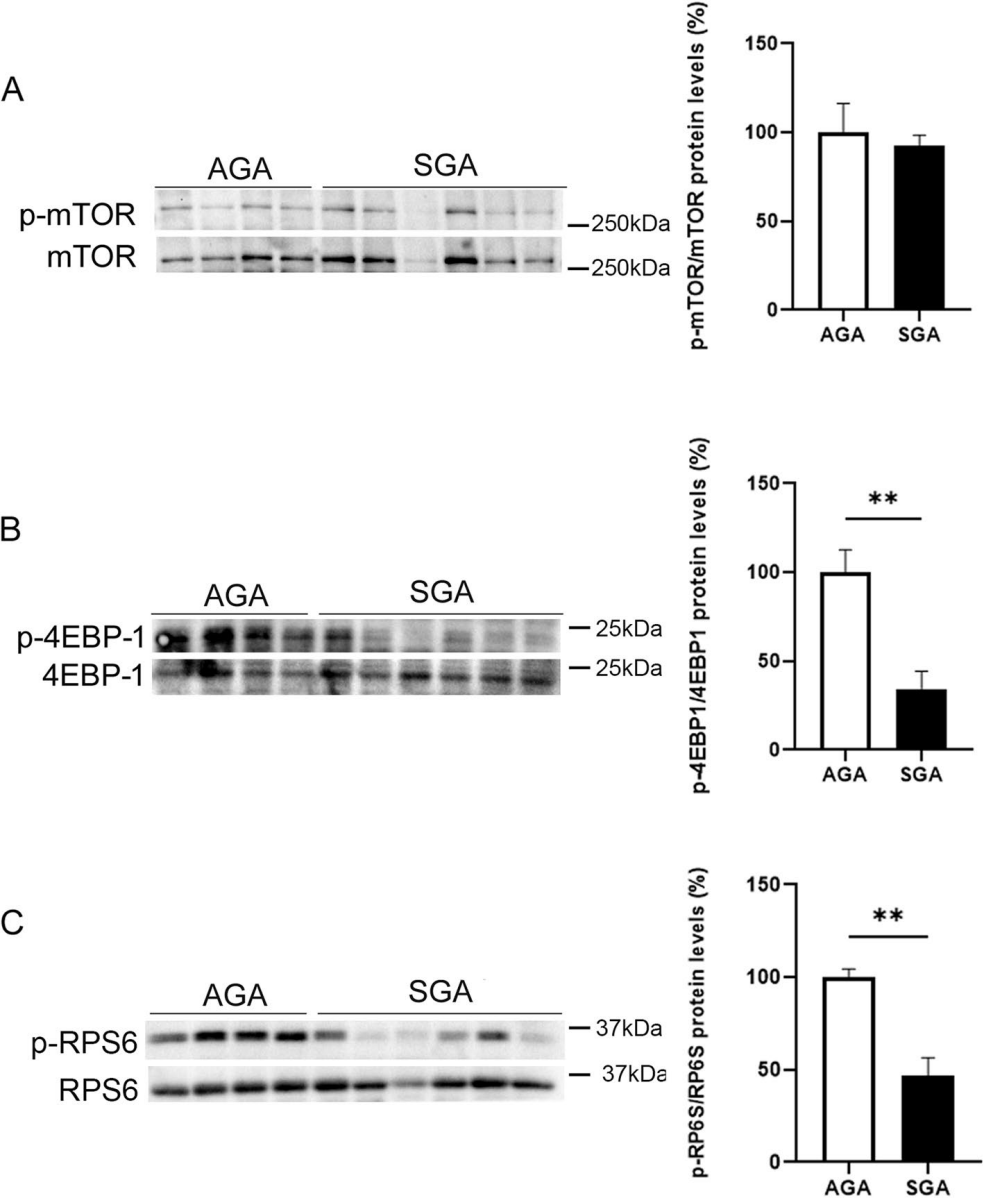
mTORC1 activity is reduced in SGA newborns. Placental cell lysate extracts were assayed via western blot analysis with antibodies against total and phosphorylated mTOR (**A**), total and phosphorylated 4EBP-1 (**B**), and total and phosphorylated RPS6 (**C**). Data (*N* = 10) are presented as the mean ± SEM. ** p < 0.01 *versus* the AGA group. *P*-values determined by two-tailed unpaired Student’s t-test

Placental mTORC1 activity was assessed by quantifying the phosphorylated levels of mTOR, 4E-BP1, and RPS6. Although mTOR phosphorylation was not different between the two groups, the phosphorylation of both 4E-BP1 and RPS6 was markedly reduced in the SGA group when compared with the AGA group (Fig. 3A-C), suggesting that the mTORC1 pathway was inhibited. By contrast, no differences were observed in the phosphorylated levels of Akt between the AGA and SGA groups, indicating that mTORC2 activity was not affected (Supplementary Fig. 1C). Overall, these findings indicate that the SGA condition is associated with increased levels of the ER stress marker CHOP and a reduction in mTORC1 activity.

### Levels of placental amino acid transporters are altered in SGA newborns

**Fig. 4:**
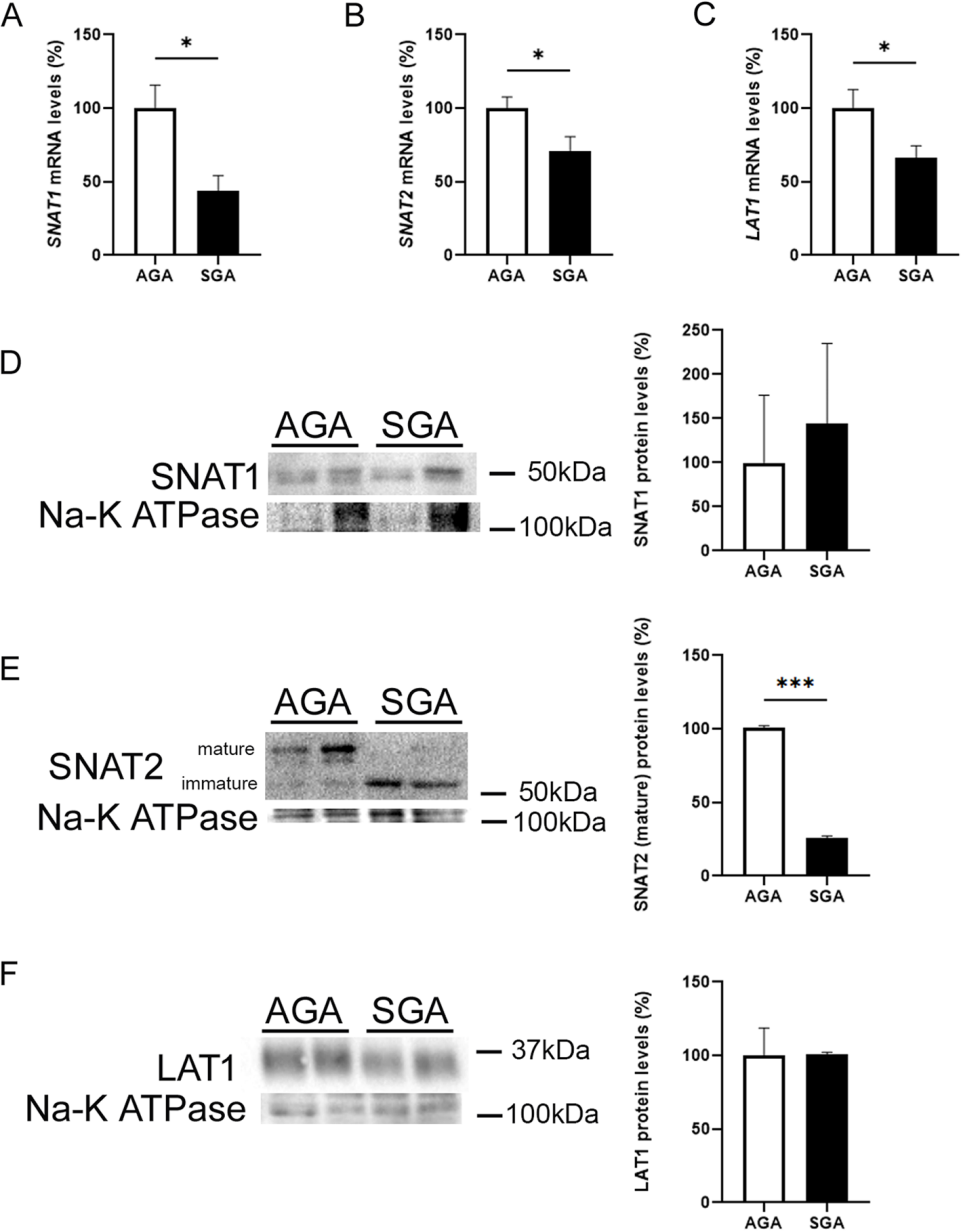
Levels of the placental mature form of the SNAT2 protein are reduced in SGA newborns. Placental mRNA levels of SNAT1 (*SLC38A1*) (**A**), SNAT2 (*SLC38A2*) (**B**), and LAT1 (*SLC7A5*) (**C**). Placental membrane cell lysate extracts were assayed via western blot analysis with antibodies against SNAT1 (**D**), SNAT2 (**E**), and LAT1 (**F**). Data (*N* = 10) are presented as the mean ± SEM. * p < 0.05 and *** p < 0.001 *versus* the AGA group. *P*-values determined by two-tailed unpaired Student’s t-test

Since a reduction in the activity of mTORC1 has been reported to inhibit trophoblast system A and system L amino acid transporter activities [20], we next evaluated whether the placentas from SGA newborns were accompanied by a reduction in the expression and protein levels of *SLC38A1* (encodes the protein SNAT1), *SLC38A2* (SNAT2), and *SLC7A5* (LAT1). Interestingly, the mRNA levels of *SLC38A1*, *SLC38A2*, and *SLC7A5* were significantly reduced in the placentas from SGA newborns when compared with the AGA group (Fig. 4A-C). When we examined the protein levels of SNAT1 in the isolated total membranes, we did not observe differences between the groups (Fig. 4D). Regarding SNAT2, it is worth mentioning that system A transporter activity depends on the processing/maturation and delivery of SNAT2 to the cell surface [28]. Antibodies against this protein detect two bands, one corresponding to the slower-migrating mature glycosylated transporter (~ 60 kDa) and the other corresponding to the faster-migrating immature SNAT2 protein that is partially processed in the ER (not or partially glycosylated, ~ 50 kDa) [29]. Interestingly, the SGA group showed a significant reduction in the levels of the mature SNAT2 protein, which was associated with increased levels of the immature form of this protein (Fig. 4E), thereby suggesting the involvement of post-transcriptional mechanisms. Finally, LAT1 protein levels were similar in both groups (Fig. 4F). Although the E3 ubiquitin-protein ligase neural precursor cell-expressed developmentally down-regulated protein 4-like 2 (NEDD4-2) (NEDD4L in humans) has been reported to be involved in the ubiquitination-mediated protein degradation of SNAT2 and LAT1 caused by mTORC1 inhibition [30], we did not observe differences in the levels of this ligase between the SGA and AGA newborns (Supplementary Fig. 1D), suggesting that this mechanism was unlikely to be involved. Since Golgi fragmentation has been reported to play a role in SNAT2 maturation [31] and given that this process is also essential for regulating the mTOR pathway [32], we examined the potential occurrence of this alteration in the placenta. Golgi re-assembly and stacking protein 65 (GRASP65) [33] and GRASP55 [34] play essential roles in the assembly and membrane stacking of the Golgi apparatus and in maintaining the Golgi structure formation. However, phosphorylation of GRASP55 has been reported to induce Golgi fragmentation [35]. We thus investigated whether the placentas from SGA newborns showed increased levels of phosphorylated GRASP55. In Phos-tag gels, the GRASP55 protein migrates as a doublet, with the upper band representing the phosphorylated form [36]. However, the placentas from SGA infants did not show differences from those of the AGA newborns (Supplementary Fig. 1E), suggesting that Golgi fragmentation is not involved in the reduced mTORC1 activity.

### GADD34 inhibition prevents the reduction of mTORC1 activity in BeWo cells

**Fig. 5:**
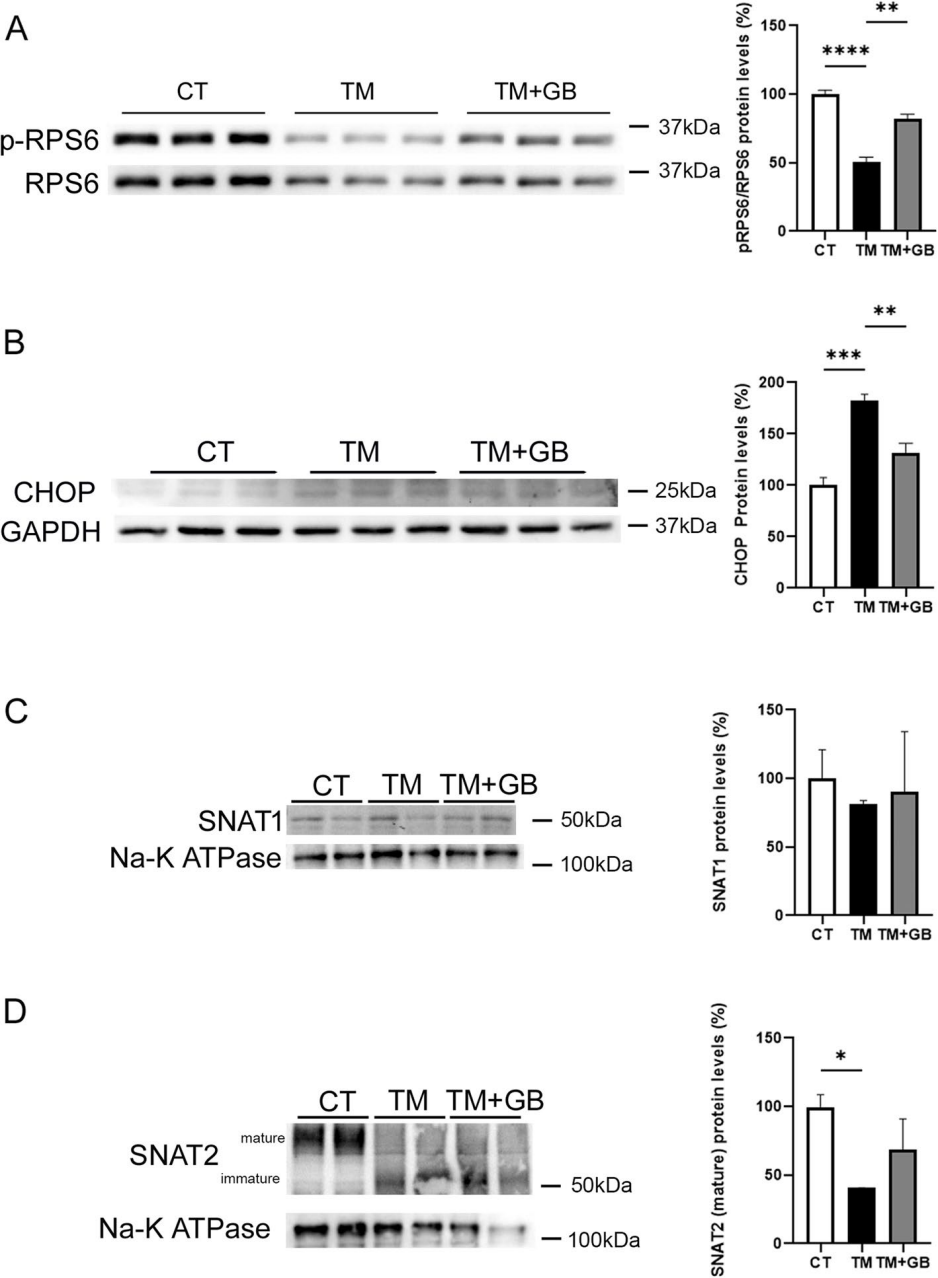
GADD34 inhibition by guanabenz attenuates the reduction in mTORC1 activity and the increase in CHOP levels caused by ER stress in the human placental cell line BeWo. BeWo cells were stimulated with 0.1 μg/ml of tunicamycin (TM) either in the presence or absence of 5 μM guanabenz (GB) for 24 h and the protein levels of total and phosphorylated RPS6 (**A**), CHOP (**B**), SNAT1 (**C**), and SNAT2 (**D**) were assessed. Data (*N* = 3) are presented as the mean ± SEM. * p < 0.05, ** p < 0.01, *** p < 0.001 and **** p < 0.0001 *versus* the AGA group. *P*-values determined by two-tailed unpaired Student’s t-test

To assess the mechanisms by which increased CHOP levels are associated with the reductions in mTORC1 activity and in the levels of amino acid transporters, we used the human placental BeWo cell line, which originated from a choriocarcinoma [37] and has been widely used as an in vitro model to study placental amino acid transporters [38, 39]. Stimulation of cells with the ER stressor tunicamycin reduced the levels of phosphorylated RPS6, indicating that mTORC1 activity was inhibited (Fig. 5A). Notably, it has been reported that GADD34 links ER stress and mTOR inactivation in the liver [26]. In fact, GADD34-knockout mice show enhanced phosphorylated mTOR signaling upon nutrient depletion, suggesting that GADD34 negatively regulates mTOR [40]. Consistent with this, incubation of the BeWo cells with tunicamycin and the GADD34 inhibitor guanabenz restored the levels of phosphorylated RPS6, thus confirming that GADD34 inhibits mTORC1 signaling. Likewise, GADD34 inhibition by guanabenz attenuated the increase in CHOP protein levels caused by tunicamycin (Fig. 5B), which is consistent with the findings of previous studies [41]. Next, we examined the effects of tunicamycin and guanabenz on the levels of SNAT1 and SNAT2. SNAT1 protein levels were not affected either by tunicamycin or guanabenz (Fig. 5C). By contrast, tunicamycin caused a marked reduction in the levels of the mature form of SNAT2 and this reduction was not restored by guanabenz, suggesting that additional mechanisms are involved in the regulation of this amino acid transporter (Fig. 5D).

## Discussion

The placenta is an organ with major endocrine and/or exocrine activity. It is more vulnerable to ER stress due to its constitutively high levels of protein translation. In fact, sustained ER stress results in compromised placental development and function [9]. Thus, it has been reported that the administration of a single dose of tunicamycin to pregnant dams causes lower placental and fetal weight, partly through placental abnormalities in nutrient transport [6]. Here, we examined placental levels of different ER stress markers in AGA and SGA newborns. A significant increase in CHOP protein levels was observed in the SGA group. The levels of GRP78 were also increased in the SGA group, while no changes were observed in the other ER stress markers. Thus, our findings suggest that low-level ER stress develops in the placentas from SGA newborns. It has been reported an increase in ER stress markers in human placenta when delivered at term by standard vaginal delivery compared to elective non-laboring caesarean section [42], with increases in the protein levels of p-eIF2α, GRP78 and X-box-binding protein 1 (XBP-1). Since caesarean sections were more frequent among SGA babies, differences in the delivery seem not to contribute to the changes in ER stress markers. The robust increase in CHOP protein levels observed in the placenta from SGA newborns might be not related to ER stress since the levels of ATF4 and ATF6, which upregulate CHOP expression under ER stress conditions, and the mRNA levels of CHOP, were not affected. The occurrence of increased CHOP levels in the placenta might be a crucial event in the reduction of birth weight by reducing mTORC1 activity, which in turn downregulates the levels of amino acid transporters. Interestingly, CHOP has been reported to upregulate GADD34 [24], which suppresses mTORC1 activity [26, 40]. Therefore, our findings suggest that the increased CHOP levels in the SGA group might contribute to the reduction in mTORC1 signaling via GADD34. mTOR is an important regulator of protein synthesis and mTOR signaling is suppressed by stressors, such as energy depletion, nutrient deprivation, and hypoxia, via the activation of tuberous sclerosis complex (TSC) 1/2. GADD34 has been reported to form a stable complex with TSC1/2, dephosphorylating TSC2, and leading to the inhibition of mTORC1 signaling [40, 43]. Consistent with this, we observed that the inhibition of GADD34 by guanabenz prevented the reduction in the mTORC1 activity caused by the ER stressor tunicamycin in BeWo cells. In addition, the reduction of the tunicamycin-mediated increase in CHOP protein levels caused by the inactivation of GADD34 by guanabenz is in accordance with the findings of a previous study conducted in HEK293T cells [41]. However, we did not observe an increase in GADD34 levels in the placentas from the SGA group compared with the AGA group. As mentioned above, GADD34 is transiently activated during ER stress [26], suggesting that this might be the reason for the absence of the increase in the levels of this protein. In line with this, a recent study has reported that GADD34 is extremely sensitive to degradation, with an estimated half-life of approximately 37 min [44]. Overall, these findings suggest that the CHOP-GADD34 pathway plays a role in the reduction of mTOR activity in the placentas from SGA newborns.

A previous study by Yung et al. reported the first evidence that placental protein synthesis inhibition and ER stress play key roles in intrauterine growth restriction (IUGR) pathophysiology [9]. This study found that decreased AKT protein reduced mTOR signaling and impaired murine placental growth. In our study we did not find changes in AKT phosphorylation in the placentas from SGA newborns. Since IUGR reflects fetal distress and SGA could represent an attenuated subtype of IUGR, these differences could be attributed to the different stages of progression of SGA and IUGR. Likewise, while Yung et al. [9] found a general induction of ER stress makers in IUGR, we only observed a robust increase in CHOP in the placentas from SGA newborns, which was accompanied by reduced mTORC1 activity and a significant decrease in the mature form of the amino acid transporter SNAT2. These findings suggest that CHOP upregulation might pull the trigger for the reduction in mTORC1 activity in the placentas from SGA newborns, while the contribution of high-grade ER stress might not be necessary. Finally, our in vitro data point to GADD34 as a potential player in the reduction in mTORC1 activity. Therefore, our findings point to the dysregulation of CHOP and of the processing/maturation of SNAT2 as key contributors to the SGA condition.

Inhibition of placental mTOR activity is associated with a reduced placental amino acid uptake and a lower birth weight [16, 45]. The SGA group exhibited a reduced expression of *SLC38A1*, *SLC38A2*, and *SLC7A5*. As mTORC1 can regulate *SLC38A2* and *SLC38A2* expression [46, 47], their reduction is likely to be the result of the inactivation of mTORC1 signaling in the placentas of the SGA group. However, the reduction in the expression of *SLC38A1* and *SLC7A5* was not accompanied by a decrease in their protein levels, suggesting that the reduction in transcription was not sufficient to attenuate their protein levels.

The regulation of *SLC38A2* transcription contributes to overall changes in SNAT2 protein levels, although post-transcriptional modifications involving the stabilization of the SNAT2 protein also play an important role [48]. A strong reduction was observed in the protein levels of the mature glycosylated SNAT2 protein in the SGA group, while the levels of the immature SNAT2 protein (not or partially glycosylated) were increased. Quantification of SNAT2 was conducted in total membrane fractions, but not in syncytiotrophoblast microvillous and basal plasma membranes, thereby preventing the study of post-translational modifications that affect the trafficking of placental SNAT2 to and from the plasma membrane. The E3 ubiquitin ligase NEDD4L has been previously implicated in the polyubiquitination and degradation of SNAT2 through the ubiquitin–proteasome system [28, 49]. However, we did not observe changes in the levels of the NEDD4L protein in the SGA group, suggesting that it was not involved in the changes observed for SNAT2. Likewise, although Golgi fragmentation may affect SNAT2 maturation [31] and controls the mTOR pathway [32], no changes were observed in the levels of phosphorylated GRASP55, a marker of this process. This therefore confirmed that Golgi fragmentation was not involved.

A limitation of this study is that it does not provide an explanation for the increase in CHOP protein in the placentas from SGA newborns independent of a high-level ER stress. Interestingly, protein N-glycosylation is a widespread post-translational modification. N-linked glycosylation is initiated in the ER and completed in the Golgi complex [50]. Alterations in glycosylation affect the proteins required for trophoblast function and have been associated with pathological conditions, including fetal growth restriction [51]. Remarkably, CHOP has been reported to be induced in the cells defective in N-glycosylation [52]. This suggests that most of the increase in the CHOP protein levels observed in the SGA newborns might be more related to defective glycosylation than to the increase in ER stress. Consistent with this, it has been reported that the increase in CHOP levels under Golgi stress is independent from the canonical UPR [53]. The strong reduction in the levels of the mature SNAT2 protein caused by the ER stressor tunicamycin in the BeWo cells seems to contradict the idea that ER stress is not the main stimulus leading to the increase in CHOP protein levels in the SGA group. However, tunicamycin is not considered a good inducer of ER stress because it blocks the assembly of N-linked glycans in the ER, causing the accumulation of the fastest migrating (immature) form of SNAT2, which is consistent with the inhibition of N-linked glycosylation [31]. Therefore, this suggests that the inhibition of glycosylation might be responsible for the reduction in the levels of the mature form of SNAT2 in the SGA group.

Another limitation of this study is that placental tissue used in our experiments contained decidua and the upper side of the chorionic villous proximal to the decidua. Since trophoblast villi (fetal tissue) and decidua (maternal tissue) are different tissues with respect to their origin, function and responses to various stimuli, the analysis of these samples may restrict the interpretation of the data.

Overall, here we unveil that the placentas from SGA newborns show low-level ER stress, with an increase in CHOP protein levels being accompanied by a reduction in mTORC1 activity and decreases in the expression of several amino acid transporters. The increase in CHOP levels contributes to the reduction of mTOR signaling in trophoblasts via GADD34. The placentas from SGA newborns also display increased levels of the immature form of SNAT2 and a consequent reduction in the levels of the mature form of this protein, which might be the result of the inhibition of N-linked glycosylation.

## Conclusions

Collectively, these results suggest that the increase in CHOP levels and the reduction in the mature form of SNAT2 contribute to a reduction in amino acid transport and, eventually, to a decreased birth weight in humans.

### Supplementary Information

**Additional file 1:** **Supplementary Figure 1.** Placental cell lysate extracts were assayed via western blot analysis with antibodies against total and phosphorylated AMPK (A), total and phosphorylated ERK1/2 (B), and total and phosphorylated Akt (C), NEDD4-L (D) and GRASP55 (E) in either a standard (bar graph quantification corresponds to this image) or a Phos-tag gel where the GRASP55 protein migrates as a doublet, with the upper band representing the phosphorylated form. The absence of a doublet in the samples from the SGA newborns as well as in the AGA newborns indicates the absence of phosphorylated GRASP55. Data (*N* = 10) are presented as the mean ± SEM.

## Data Availability

The source data for this study are available as a Source Data file or from the corresponding author upon reasonable request.
