# Supplementary figures and images for: CHOP upregulation and dysregulation of the mature form of the SNAT2 amino acid transporter in the placentas from small for gestational age newborns
Source: Cell Commun Signal. 2023 Nov 13;21:326. doi: 10.1186/s12964-023-01352-5 (PMC10644500; doi:10.1186/s12964-023-01352-5)

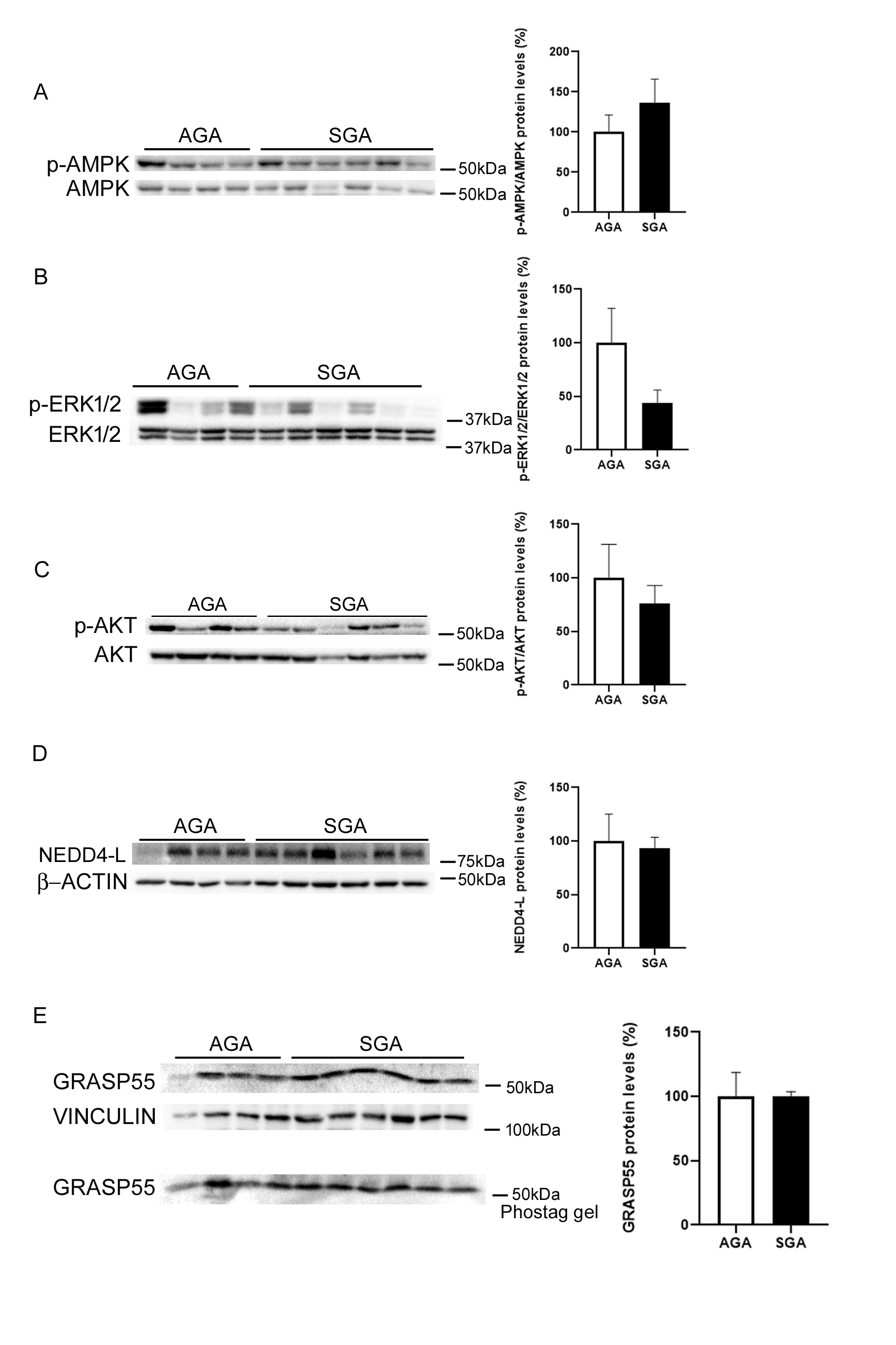

Supplement: Supplementary file 2 — Additional file 1: Supplementary Figure 1. Placental cell lysate extracts were assayed via western blot analysis with antibodies against total and phosphorylated AMPK (A), total and phosphorylated ERK1/2 (B), and total and phosphorylated Akt (C), NEDD4-L (D) and GRASP55 (E) in either a standard (bar graph quantification corresponds to this image) or a Phos-tag gel where the GRASP55 protein migrates as a doublet, with the upper band representing the phosphorylated form. The absence of a doublet in the samples from the SGA newborns as well as in the AGA newborns indicates the absence of phosphorylated GRASP55. Data (N = 10) are presented as the mean ± SEM. [file 12964_2023_1352_MOESM1_ESM.tif]
